# Integration of Family Navigation into ECHO Autism for Pediatric Primary Care in Underserved Communities

**DOI:** 10.1007/s10803-024-06445-9

**Published:** 2024-07-02

**Authors:** Micah O. Mazurek, Rose E. Nevill, Karen Orlando, Keith Page, Mya Howard, Beth Ellen Davis

**Affiliations:** 1 Department of Human Services, School of Education and Human Development, University of Virginia, 417 Emmet Street South, PO Box 400267, Charlottesville, VA 22904 USA; 2 Division of Neurodevelopmental and Behavioral Pediatrics, Department of Pediatrics, University of Virginia, Charlottesville, VA USA

**Keywords:** Autism, Primary care provider, Screening, Family navigation, Healthcare barriers, Care coordination

## Abstract

Children with autism from underserved communities face complex system-, provider-, and family-level barriers to accessing timely diagnosis and early intervention. The current study evaluated the preliminary effects and feasibility of a new program (ECHO Autism LINKS) that integrated pediatric primary care provider (PCP) training with family navigation (FN) to bridge the gaps between screening, referral, and service access. Three cohorts of PCPs (*n* = 42) participated in the program, which consisted of 60-minute sessions delivered by Zoom twice per month for 12 months. Each session included didactics, case-based learning, and collaborative discussion with participants and an interdisciplinary team of experts. Family navigators were members of the expert team and provided FN services to families referred by PCP participants. Program attendance and engagement were strong, with 40 cases presented and 258 families referred for FN services, most of whom (83%) needed help accessing and connecting with services, and 13% required ongoing support due to complex needs. PCPs demonstrated significant improvements in self-efficacy in providing best-practice care for children with autism, reported high satisfaction, and observed improved knowledge and practice as a result of the program. The results of this initial pilot provide support for the feasibility, acceptability, and preliminary efficacy of the ECHO Autism LINKS program. The model holds promise in addressing complex barriers to healthcare access by providing both PCPs and families with the knowledge and support they need. Future research is needed to evaluate the efficacy and effectiveness of the program in improving child and family outcomes.

Autism prevalence has steadily increased over the past several decades, with the most recent estimates from the Centers of Disease Control and Prevention (CDC) indicating that 1 in 36 children in the United States (US) meets criteria for autism spectrum disorder (ASD) (Maenner et al., 2023). As a result, there is an ever-growing demand for high-quality pediatric healthcare for children with autism and their families. Unfortunately, many children with autism have significant unmet healthcare needs and experience worse access to family-centered, coordinated, and comprehensive care than children with other disabilities (Karpur et al., 2019; Vohra et al., 2014). Although autism can be reliably detected and diagnosed in the first two years of life (Zwaigenbaum et al., 2015), the average age at autism diagnosis in the US ranges from 3 to 5 years of age (Hanley et al., 2021; Maenner et al., 2023), with considerable delays between caregivers’ first concerns and ultimate diagnosis (MacKenzie et al., 2022). These delays have negative cascading effects on timely access to appropriate early intervention services, which are important for promoting optimal developmental outcomes for autistic children (Sandbank et al., 2020; Zwaigenbaum et al., 2015). Unfortunately, children from under-resourced and marginalized communities face even greater disparities in access to care (Liu et al., 2023).

## Barriers to Early Identification & Intervention

The barriers that contribute to these unmet healthcare needs are multifold. First, there are longstanding shortages of healthcare providers specializing in autism (Bridgemohan et al., 2018; Cantor et al., 2020; McBain et al., 2020). The growing demand for services has far outpaced the capacity of these specialists, resulting in a waitlist crisis for autism services across the US (Jimenez et al., 2017; Kanne & Bishop, 2021). Autism resources are also unevenly distributed across the country, with most resources clustering near academic medical centers and in affluent areas (Drahota et al., 2020). For example, an analysis of data derived from GapMap (a comprehensive autism resource database) estimated that 84% of all US counties lack autism diagnostic resources (Ning et al., 2019). As a result, families from rural and under-resourced communities are often required to travel considerable distances to access care (Gresenz et al., 2006; Mello et al., 2016).

To address these challenges, recent efforts have focused on promoting early identification of autism in primary care settings. The American Academy of Pediatrics (AAP) has provided clear and specific guidance for autism screening and management, including systematic and universal surveillance, general developmental screening, and autism-specific screening at regular well-child visits (Hyman et al., 2020; Myers & Johnson, 2007). However, pediatric primary care physicians and practitioners (PCPs) have struggled to consistently implement these recommendations, with recent studies showing generally poor PCP adherence to AAP recommendations for developmental and autism screening (Lipkin et al., 2020; Mazurek, Kuhlthau, et al., 2021; Rea et al., 2019).

Even when pediatric providers appropriately identify early signs of autism, many are not making referrals for recommended services, despite clear referral guidance from the AAP (Hyman et al., 2020). For example, in a study of 290 PCPs across 54 pediatric practices, only 31% of children with positive autism screens were referred for further evaluation (Monteiro et al., 2019). In a separate study of 999 children seen in an urban pediatric primary care setting, only 27% of children with positive screens were referred for audiology and only 57% were referred for early intervention (Rea et al., 2019). Within a different pediatric primary care network, of 2,882 children who screened positive on the M-CHAT-R, only 40% received at least one recommended referral (Wallis et al., 2020).

Real-world challenges in adhering to best-practice clinical guidelines are likely the result of multiple intersecting factors. In general, PCPs report a lack of prior training in identification, diagnosis, and medical care of children with autism (Mazurek, Harkins, et al., 2020; Self et al., 2015), and many PCPs lack specific knowledge of autism screening tools and practices (Carbone et al., 2013; Fenikilé et al., 2015). In a study of 114 PCPs practicing across 14 states, most (81%) reported that their lack of knowledge of autism resources was a key barrier to caring for autistic patients, and many felt that a better understanding of community service systems would be particularly helpful (Mazurek, Harkins, et al., 2020). In another qualitative study, PCPs expressed uncertainty about autism resources and services that led them to avoid making referrals (Hamp et al., 2023). In another study, pediatric PCPs reported taking a “wait and see” (29%) or “wait and evaluate further” (55%) approach after noting developmental delays among toddlers. The majority (52%) felt that additional training on autism and the referral process would help promote earlier referrals (Edwards et al., 2021). As a result of these provider-level factors, many children with autism experience barriers at each point along the pathway from first concern to referral for services, significantly reducing access to critical interventions and resources.

Unfortunately, even when PCPs make timely referrals, many families struggle to follow-through with their providers’ recommendations. Children with autism often require care from multiple professionals across a wide range of healthcare, mental health, educational, and community-based agencies (Pumariega, 2022), which often have complex and variable eligibility and funding requirements (Miller et al., 2016). These systems are also often fragmented and disconnected, leaving it up to families to locate, coordinate, and navigate care for their children (Brewer, 2018; Lappé et al., 2018). This process can be daunting and confusing. As a result, many children do not receive recommended services, even after provider referrals are made (Karp et al., 2018; Sapiets et al., 2021; Warren et al., 2013). Families from rural, marginalized, and underserved populations face even worse access to autism-related services (Karp et al., 2018; Liu et al., 2023; Smith et al., 2020).

## Approaches for Improving Access to Care

Efforts to improve access to timely care for children with autism have ranged from provider-focused to family-focused. High-level provider-focused strategies have included resources from professional organizations, such as autism-focused AAP clinical guidelines (Hyman et al., 2020; Myers & Johnson, 2007) and the CDC’s “Learn the Signs. Act Early” program (Centers for Disease Control and Prevention, 2023). Other efforts have focused on pre-professional education, such as the “Autism Case Training: A Developmental-Behavioral Pediatrics Curriculum’’ developed for pediatric residency programs in partnership with the CDC and Maternal and Child Health Bureau (Major et al., 2013).

Other provider-level approaches have focused on building capacity within the current workforce through post-professional training in autism. For example, the Screening Tools and Referral Training-Evaluation and Diagnosis (START-ED) program provides a 2-day workshop for PCPs on autism screening and standardized observational assessment (Swanson et al., 2014; Warren et al., 2009). Other programs have offered in-person workshops (Bordini et al., 2014; Eray & Murat, 2017), or have combined full- or half-day workshops with follow-up support through conference calls (Carbone et al., 2016).

Another model, the ECHO Autism program, leverages technology to provide ongoing training and mentorship for PCPs in best-practice care for children with autism (Mazurek et al., 2017; Sohl et al., 2017). The ECHO Autism program establishes an ongoing learning community by connecting community-based PCPs with one another and with an interdisciplinary expert team. Sessions are held regularly via videoconference, and include didactics, case-based learning, and collaborative mentorship. Previous studies of ECHO Autism have found specific improvements in PCP self-efficacy (Malow et al., 2023; Mazurek et al., 2017, 2019; Mazurek, Parker, et al., 2020, Mazurek, Stobbe, et al., 2020), knowledge (Malow et al., 2023; Mazurek et al., 2017, 2019; Mazurek, Parker, et al., 2020; Mazurek, Stobbe, et al., 2020), and competence in autism screening and diagnosis (Bellesheim et al., 2020; Mazurek et al., 2019; Schieltz et al., 2023; Sohl et al., 2023). In addition, participation in ECHO Autism has resulted in significant improvements in PCP confidence in their ability to identify and refer to autism-specific community resources and services (Malow et al., 2023; Mazurek et al., 2017, 2019; Mazurek, Stobbe, et al., 2020).

Beyond provider-level knowledge and skill, an equally important consideration for promoting children’s access to care is whether families follow through on referrals and connect with recommended resources. Given the complexity and fragmentation of autism-related service systems, many families need help coordinating and navigating care (Carbone et al., 2010; Fenikilé et al., 2015; Mazurek, Harkins, et al., 2020; Mazurek, Sadikova, et al., 2021). Family navigation (FN) models represent one particularly promising approach for providing these supports. The FN approach is an adaptation of the patient navigation (PN) model, which was originally developed to support patients with cancer by offering information, emotional support, and system navigation (Freeman et al., 1995; Palos & Hare, 2011), and has more recently been expanded to focus on families of children with autism (Broder-Fingert et al., 2020; Gardiner et al., 2022). Key components of these programs include identifying individual needs and barriers, providing information and emotional support, facilitating coordination of resources and services, and utilizing a family-centered and collaborative approach (Broder-Fingert et al., 2020; Gardiner et al., 2022).

There is a growing body of literature demonstrating the effectiveness of FN in facilitating access to care for children with autism. For example, in a randomized study of 250 families whose toddler screened positive for autism, those receiving FN services had a greater likelihood of accessing diagnostic assessments than those receiving conventional care management (Feinberg et al., 2021). Similarly, in a pragmatic randomized trial, families who received FN services were more likely to access an autism diagnostic evaluation compared to those in the usual care condition (DiGuiseppi et al., 2021). In another randomized study of newly diagnosed children with autism, families who received early FN services were more successful in obtaining recommended services for their children (Roth et al., 2016). As a whole, these studies suggest that FN is a promising approach that may close the gaps between referral and access to services, particularly for under-resourced families.

## Current Study

Given these complex barriers to early identification and early intervention, innovative approaches are needed to address system-, provider-, and family-level barriers to healthcare access. Equipping PCPs with greater knowledge and self-efficacy regarding autism identification and management *and* providing families the support they need to navigate care may help bridge the gaps between screening, referral, and service access. The purpose of this study was to evaluate the preliminary effects and feasibility of an adapted ECHO Autism program that integrated both PCP training and FN services to enhance access to early identification and intervention. This program was called ECHO Autism LINKS (Leading Innovation through Navigation, Knowledge and Supports).

## Methods

### Participants

Pediatric primary care physicians and practitioners (PCPs) from across Virginia were recruited for participation, with a targeted focus on underserved regions of the state. Eligibility included providing primary care to children (e.g., general pediatricians, family medicine physicians, nurse practitioners, or physician assistants). Recruitment strategies included direct outreach to clinics in targeted rural and underserved areas of the state by phone, email, and word of mouth. Flyers were also shared with community agencies, including those funded through the Title V Maternal and Child Health Block Grant, and through professional listservs, including the state chapter of the American Academy of Pediatrics (AAP) and the state Council of Nurse Practitioners. The study was approved by the Institutional Review Board at the University of Virginia, and all participants provided informed consent. Three consecutive cohorts of PCPs participated in the ECHO Autism LINKS program, resulting in a total sample of 42 PCP participants (*n* = 14 in Cohort 1, *n* = 15 in Cohort 2, and *n* = 13 in Cohort 3).

### ECHO Autism LINKS Program

The ECHO Autism LINKS program was designed to provide training and mentorship for pediatric PCPs in best-practice care for children with autism along with integrated FN support for families. The ECHO Autism LINKS curriculum was adapted from the original ECHO Autism program, with incorporation of additional elements to focus more specifically on facilitating access to early intervention and coordinated care. The original ECHO Autism program consisted of two-hour sessions delivered twice per month over a six-month period (Mazurek et al., 2017; Mazurek, Parker, Mazurek et al., 2020a, b, c). The newly adapted program expanded the curriculum to a 12-month program of 60-minute sessions delivered twice per month. Consistent the with the ECHO framework (Arora et al., 2007, 2016), sessions were held via Zoom videoconferencing, and participating PCPs (“spokes”) were connected with one another and an interdisciplinary expert team (“hub”). Each session included introductions, a brief didactic presented by a member of the hub team, and a de-identified case presented by one of the PCP participants for discussion and guidance. Participants were encouraged to keep their cameras on during the session to facilitate a greater sense of community and engagement. The interdisciplinary hub team included a developmental-behavioral pediatrician, a child and adolescent psychiatrist, a clinical psychologist, and a speech-language pathologist. The hub team also included two family navigators, who presented didactics, contributed to case discussions, developed electronic FN resources, and provided FN services to families referred by PCP participants. Both family navigators held master’s degrees (Master of Science in Education in Intensive Special Needs, and Master of Teaching, respectively) and had professional experience providing FN to families of children with autism. In addition, one had prior experience as an elementary school autism specialist and as a consultant for families of children with autism, and one brought additional lived experience as the parent of a child with autism.

The didactic curriculum focused on best-practice guidelines for screening and evidence-based care for children with autism, with a particular focus on early childhood and access to care. Topics are shown in Table 1. Similar to previous adaptations of the ECHO Autism model (Bellesheim et al., 2020; Mazurek et al., 2019), every 4th session, PCPs participated in learning network discussions focused on strategies for (1) screening during well-child visits, and (2) streamlining referrals to EI, community-based services, and specialty care. These sessions incorporated quality improvement (QI) principles for continuous monitoring and small tests of change, and were framed after the Institute for Healthcare Improvement’s Breakthrough series (Kilo, 1998). The “Plan, Do, Study, Act” (PDSA) cycle concept was used to help PCPs plan, implement, and evaluate ideas for practice change. Between sessions, PCPs tracked their own screening and referral practices. During each learning network discussion, aggregate data were shared, and PCPs collaborated on ideas for continued refinement of clinical care processes for screening and referral. Participants were eligible for both Maintenance of Certification (MoC) (Parts 2 and 4) and Continuing Medical Education (CME) credits (Category 1). Twenty-nine participants received CME credits, 13 received MoC Part 2 credits, and 16 received MoC Part 4 credits.

**Table 1:** ECHO autism LINKS curriculum

Didactic topics	Percent of session attendees rating didactic as helpful to practice
Autism Overview	69.6%
Developmental Screening	78.3%
Standardized Screening^a^	77.8%
Autism Screening	73.9%
Diagnostic Assessment	72.0%
Early Intervention	72.0%
Parent Support	77.3%
Community Resources	88.9%
Waivers & Financial Resources^b^	85.7%
Successful Clinic Visits	60.0%
Communication Strategies	73.9%
Educational Supports	72.2%
Behavioral Strategies	78.3%
Managing ADHD	73.7%
Managing Irritability	82.6%
Managing Anxiety	90.5%
Managing Sleep	87.5%
Feeding/Gastrointestinal Concerns	90.0%
Transition to Adulthood^c^	85.7%

*Note* Some topics were included by demand for certain Cohorts

^a^ Included in Cohort 1

^b^ Included in Cohorts 2 & 3

^c^ Included in Cohorts 1 & 3

Family navigation was integrated throughout the ECHO Autism LINKS program. In their role on the hub team, family navigators provided didactic training for PCPs in principles and practices of family-centered care, parent-provider partnerships, community service systems and resources, and care coordination. They also offered expertise, guidance, and recommendations during case discussions. In this context, family navigators provided information about specific community services and resources, as well as offering insight into effective strategies for family engagement, overcoming barriers to follow-through, and facilitating access to care. In between sessions, FN hub team members worked to create regional resource roadmaps and toolkits for families (e.g., annotated resource lists, step-by-step guides, topic- and age-based infographics, etc.). These materials were distributed to PCP participants (via email and on a shared online drive) to be shared with families in their clinics. In addition, PCP participants were able to make direct referrals to family navigators on the hub team for follow-up support. After receiving a referral, the navigator completed a phone intake to determine whether the family needed one-time support (sharing referral information, resource lists, or information) or ongoing navigation and/or care coordination.

### Measures

Participants completed online questionnaires at pre-training (prior to attending the first ECHO Autism LINKS session) and post-training (after completion of the 12-month ECHO Autism LINKS program). Pre- and post-data were de-identified and linked using ID codes. De-identified case presentation forms, participant attendance, and FN referral forms were also examined.

#### Demographic Survey

Participants completed a brief demographic survey at baseline (pre-training). Information included age, gender, race, ethnicity, professional discipline, years in practice, current practice setting, zip code of current practice, previous training in autism, and reasons for interest in participating in the program.

#### Self-Efficacy Survey

Self-efficacy was assessed at both pre- and post-training using a slightly adapted version of the Primary Care Autism Self-Efficacy (PCASE) Survey (Mazurek et al., 2017). Adaptations for the current study included removing some items not relevant to the curriculum, resulting in a reduction in the total number of items from 57 to 51. On this survey, participants are asked to rate their degree of confidence in their ability to effectively provide specific aspects of care, with item ratings ranging from 1 (*no confidence*) to 6 (*highly confident/expert*). The survey generates both a Total Score (sum of all items) and subscale scores within the following domains: autism screening and identification (6 items), autism referral and resources (9 items), assessment and treatment of co-occurring medical conditions (14 items), assessment and treatment of psychiatric symptoms (13 items), and additional aspects of care for autism (9 items). Subscale scores are calculated as the mean score across items within each domain to facilitate comparisons across subscales with different numbers of items.

#### Satisfaction Survey

After participation in the ECHO Autism LINKS program (post-training), participants completed a 25-item survey to share their overall perceptions of the program. The survey included 10 items rated on a 5-point scale (1 = *strongly agree*; 5 = *strongly disagree*), with lower overall scores reflecting greater satisfaction. Additional items focused on perceptions of specific aspects of the ECHO program (e.g., session length, use of the resources library, case-based learning, case presentation form, and didactic topics).

### Data Analysis Plan

Sample characteristics, characteristics of de-identified case presentations, and FN referral data were examined using descriptive statistics (i.e., mean, standard deviation, range, percentage). Paired-samples t-tests were used to determine whether there were statistically significant changes in self-efficacy from pre- to post-training. Open-ended text responses describing reasons for referral and primary concerns were evaluated using qualitative methods. Two authors (MM and MH) reviewed all written responses, formulated initial impressions of the data, and employed a constant comparative approach for identification of initial categories (Boeije, 2002). These initial impressions were discussed and compared by both authors until consensus was reached for final categories and codes. These categories were then discussed and reviewed by additional members of the ECHO and FN team to ensure trustworthiness of the results.

## Results

### Characteristics of PCP Participants

A total of 42 PCPs participated in the ECHO program. Participant characteristics are shown in Table 2. Most participants (66.7%) were general pediatricians, and most participants (73.8%) had not received any previous training focused on autism. The most common reasons for interest in participating in the program were a desire to “learn more about autism/developmental disabilities” (92.9%), “be more comfortable with complex behavioral and medical conditions associated with autism/developmental disabilities” (90.5%), and “learn more about appropriate autism/developmental disability resources” (88.1%). Participant attendance ranged from 4.17 to 100% of all ECHO sessions, with an average attendance of 67.6% (*SD* = 29.23%; median = 76.4%; mode = 83.3%) across participants.

**Table 2:** Characteristics of PCP participants

	Total Sample (*n* = 42)	Cohort 1 (*n* = 14)	Cohort 2 (*n* = 15)	Cohort 3 (*n* = 13)
	*M* (*SD*)	*M* (*SD*)	*M* (*SD*)	*M* (*SD*)
Age	46.1 (10.8)	48.0 (11.1)	42.7 (11.1)	47.5 (10.1)
Years of Practice	14.9 (9.0)	15.7 (10.5)	12.1 (8.7)	17.3 (10.5)
	*n* (%)	*n* (%)	*n* (%)	*n* (%)
Gender				
Male	7 (16.7)	2 (14.3)	2 (13.3)	3 (23.1)
Female	35 (83.3)	12 (85.7)	13 (86.7)	10 (76.9)
Race/Ethnicity				
American Indian or Alaska Native	1 (2.4)	0 (0)	1 (6.7)	0 (0)
Asian	2 (4.8)	0 (0)	1 (6.7)	1 (7.7)
Black or African American	4 (9.5)	1 (7.1)	3 (20.0)	0 (0)
Hispanic or Latino	0 (0)	0 (0)	0 (0)	0 (0)
White or Caucasian	35 (83.3)	13 (92.9)	11 (73.3)	11 (84.6)
Professional Discipline				
General Pediatrician	28 (66.7)	9 (64.3)	10 (66.7)	9 (69.2)
Internal Medicine-Pediatrics (Med-Peds) Physician	3 (7.1)	2 (14.3)	0 (0)	1 (7.7)
Family Medicine Physician	1 (2.4)	1 (7.1)	0 (0)	0 (0)
Nurse Practitioner	9 (21.4)	2 (14.3)	4 (26.7)	3 (23.1)
Physician Assistant	1 (2.4)	0 (0)	1 (6.7)	0 (0)
Practice Type				
Private Practice	21 (50)	10 (71.4)	5 (33.3)	6 (46.2)
Federally Qualified Health Center	9 (21.4)	3 (21.4)	4 (26.7)	2 (15.4)
Hospital Owned Practice	4 (9.5)	3 (21.4)	0 (0)	1 (7.7)
University Affiliated Clinic	4 (9.5)	2 (14.3)	1 (6.7)	1 (7.7)
Other	4 (9.5)	2 (14.3)	0 (0)	2 (15.4)

### Cases Presented

Twenty-seven PCP participants (64.3% of the sample) presented at least one case for discussion during an ECHO session, with 10 (23.8%) presenting more than one case over the course of the ECHO program. Across all three cohorts, a total of 40 de-identified cases were presented for discussion and guidance. Cases ranged in age from 15 months to 17 years (*M* = 5.41 years, *SD* = 4.11) and were mostly male (63%). See Table 3 for more detail regarding case characteristics. The most common consultation question across cases was autism screening and diagnostic clarification (65% of cases), followed by questions about appropriate community-based resources and services (55% of cases). In 27.5% of cases, PCPs were specifically seeking support in how to effectively partner with families to facilitate follow-through on recommendations and referrals.

**Table 3:** Characteristics of cases presented (*n* = 40)

	*M (SD)*
Age	5.41 (4.11)
Age at Autism Diagnosis^a^	2.83 (1.3)
	*n (%)*
Sex	
Male	25 (62.5)
Female	15 (37.5)
Race/Ethnicity	
Black or African American	4 (10.0)
Hispanic or Latino	3 (7.5)
White or Caucasian	32 (80.0)
Other/Unknown	3 (7.5)
Insurance Type	
Medicaid	19 (47.5)
Private Insurance	19 (47.5)
Unknown	2 (5.0)
Primary Consultation Questions/Concerns	
Autism Screening/Identification	26 (65.0)
Management of Symptoms	21 (52.5)
Resources and Services	22 (55.0)
Family-Professional Partnership	11 (27.5)
Additional Concerns	
Aggression	13 (32.5)
Self-injurious Behavior	5 (12.5)
Elopement/Wandering	6 (15.0)
Attention Problems and/or Hyperactivity	17 (42.5)
Anxiety	10 (25.0)
History of Trauma	7 (17.5)
Sleep Problems	12 (30)
Chronic Gastrointestinal Problems	10 (25.0)
Neurological symptoms (seizures, staring spells)	3 (7.5)

^a^Among the subsample of *n* = 14 with a pre-existing diagnosis

### Family Navigation Referrals

Over the course of the project, 258 families were referred for FN services by participating PCP clinics. See Table 4 for characteristics of cases referred. Of those, only 21 families (8.1% of those referred) did not respond after multiple attempts by the FN team to contact them and initiate services. Of those referred for FN services, over half (65%) had also been referred for an evaluation to rule-out autism, and many had been referred to multiple services (including speech-language therapy, occupational therapy, physical therapy, applied behavior analysis, audiology, and others). Most cases (71.7%) had public insurance (i.e., Medicaid or Managed Medicaid).

**Table 4:** Characteristics of cases referred for Family Navigation (*n* = 258)

		*M (SD)*
Child Age		5.88 (4.38)
		*n (%)*
Race/Ethnicity		
Asian		2 (0.8)
Black or African American		49 (19.0)
White or Caucasian		111 (43.0)
Multiracial		20 (7.8)
Hispanic or Latino		21 (8.1)
Not reported		61 (23.6)
Language Spoken in Home (other than English)		
Spanish		17 (6.6)
Arabic		3 (1.2)
Other		5 (1.9)
Primary Insurance		
Public (Medicaid or Managed Medicaid)		185 (71.7)
Private		57 (22.1)
Military (Tricare)		4 (1.6)
Uninsured		3 (1.2)
Not reported		9 (3.5)
Current Diagnosis at time of FN Referral		
Autism		143 (55.4)
Developmental Delay		96 (37.2)
Speech/Language Delay		122 (47.3)
Motor Delay		22 (8.5)
ADHD		55 (21.3)
Intellectual Disability		17 (6.6)
Other		77 (29.8)
Referrals for Additional Services		
Autism Diagnostic Evaluation		169 (65.5)
Speech-Language Therapy		93 (36.0)
Occupational Therapy		54 (20.9)
Physical Therapy		28 (10.9)
Applied Behavior Analysis		56 (10.9)
Audiology		43 (16.7)
Other		61 (23.6)

Regarding primary reasons for FN referral, most families (83%) were referred for assistance with accessing and connecting with services, the most common being developmental-behavioral pediatrics (30%) and ABA (27%). Some families needed access to information (19%), while others were referred for more extensive care coordination (11%). Many families (21%) were referred for family support to address specific needs, including financial (5%), family stressors (8%), managing behavior and safety in the home (11%), and managing sleep problems (3%). In some cases, families who were new to the area (7%) needed assistance with identifying local resources and services. Initial FN determination regarding level of support required indicated that 24% required one-time support (sharing referral information, resource lists, or information), while most families required ongoing support, with approximately 13% requiring high-level, long-term FN support due to complex psychosocial needs.

### PCP Self-Efficacy

Of the 42 PCP participants, 14 did not complete surveys at both time points, resulting in a sample size of 28 for analyses of pre- to post-training change. Overall, total self-efficacy raw scores improved significantly from pre-training (*M* = 180.36, SD = 27.99) to post-training (*M* = 265.18, SD = 29.19), *t*(27) = -13.47, *p* < .001. Statistically significant improvements were also observed across all self-efficacy subdomains, including autism screening and identification [*t*(27) = -8.21, *p* < .001], autism referral and resources [*t*(27) = -11.75, *p* < .001], assessment and treatment of co-occurring medical conditions [*t*(27) = -12.20, *p* < .001], assessment and treatment of psychiatric symptoms [*t*(27) = -12.00, *p* < .001], and additional aspects of care for autism [*t*(27) = -11.73, *p* < .001].

### PCP Perceptions and Satisfaction with the Program

Participant satisfaction was high across all items (ratings on a 5-point scale with “1” indicating the highest degree of satisfaction). Specifically, participants reported that the ECHO Autism LINKS program helped them improve their ability to care for children with autism in their practice (*M* = 1.07, *SD* = 0.27), that they learned best-practice care for autism (*M* = 1.15, *SD* = 0.36), and that they were more confident in referring families of children with autism to community-based supports (*M* = 1.19, *SD* = 0.40). Participants were satisfied with the technology associated with the program (*M* = 1.19, *SD* = 0.51), and with the guidance received from the ECHO Autism hub team (*M* = 1.07, *SD* = 0.27). They also reported that specific elements of the ECHO program enhanced their knowledge about autism, including didactic presentations (*M* = 1.15, *SD* = 0.27), discussions with other participants (*M* = 1.26, *SD* = 0.53), and case-based learning (*M* = 1.22, *SD* = 0.51). Most respondents reported that they had used the ECHO Autism LINKS resource library (80.7% agreed or strongly agreed) and that they were likely to use it in the future (96.3% agreed or strongly agreed). All participants (100%) reported that they would be likely to recommend the ECHO Autism LINKS program to colleagues in the future. The perceived helpfulness of each didactic topic is shown in Table 1, as indicated by the percentage of attendees (per session) who rated the didactic as helpful to them or their practice.

## Discussion

Children with autism and their families face complex system-, provider-, and family-level barriers to accessing both diagnostic and intervention services. There are shortages of healthcare providers specializing in autism (Bridgemohan et al., 2018; Cantor et al., 2020; McBain et al., 2020), and many PCPs lack knowledge and training in autism screening and evidence-based care (Fenikilé et al., 2015; Mazurek, Harkins, et al., 2020). This contributes to significant delays in autism identification, diagnosis, and intervention for many children (MacKenzie et al., 2022). Compounding these issues, many families struggle to follow-through on referrals due to the complex and fragmented nature of the autism service system (Karp et al., 2018; Sapiets et al., 2021; Twardzik et al., 2017). The current study piloted a new integrated approach for addressing these barriers. The ECHO Autism LINKS program combined two well-established models for improving healthcare access: the ECHO framework (for training providers in screening, referral, and family-centered care) and the FN approach (for supporting families in navigating and coordinating care). The results of this initial pilot provide support for the feasibility, acceptability, and preliminary efficacy of this integrated program.

PCPs participating in the program reported high satisfaction and demonstrated significant improvements in their confidence in providing evidence-based care for children with autism. This ranged from improved self-efficacy in autism screening and identification, to greater perceived ability in providing comprehensive ongoing support and referrals. This is an important finding, particularly since low self-efficacy has been repeatedly identified as a critical provider-level barrier to care for children with autism (Carbone et al., 2013; Mazurek, Harkins, et al., 2020; Self et al., 2015). These results are also highly consistent with findings from previous studies of ECHO Autism (Mazurek et al., 2017, 2019; Mazurek, Parker, et al., 2020). Overall, the combination of didactics and ongoing mentoring through a community of practice appears to be highly successful in equipping PCPs with the knowledge and confidence to deliver evidence-based care. In fact, in a previous qualitative study, PCPs participating in ECHO Autism observed that a sense of community and the expertise of the hub team were key complementary benefits of the program (Cheak-Zamora et al., 2021). Additionally, participating in specific QI-based learning network activities (similar to Bellesheim et al., 2020; Mazurek et al., 2019), likely further accelerated practice change in the areas of screening and referral to early intervention.

An examination of cases presented by PCPs for discussion during ECHO sessions indicates that over half (65%) were focused on autism screening and identification. In addition, PCPs also requested support in managing co-occurring symptoms (52% of cases) and identifying resources and services (55% of cases). This is not surprising, given the high rates of co-occurring medical and psychiatric conditions among children with autism (Micai et al., 2023) and their associated need for specialized services and supports (Hyman et al., 2020). Results from both the self-efficacy and satisfaction surveys suggest that the ECHO Autism LINKS program was effective in building knowledge and confidence among PCPs in addressing these healthcare needs and referring children to community-based services and supports.

Interestingly, PCPs requested specific guidance on building effective family-professional partnerships in several cases presented (27%). This speaks to the need for a dual approach for equipping both providers and families with the support they need to effectively facilitate family-centered and comprehensive care for children with autism. In fact, a unique element of the ECHO Autism LINKS program was the integration of FN throughout the program. In their role on the ECHO Autism hub team, family navigators provided direct training and guidance for PCPs in effective strategies for family engagement and partnership. They also developed a library of local resources and roadmaps that PCPs could share with families. By the end of the program, almost all PCP participants (96%) planned to continue to use the resource library in the future.

Most notably, PCPs referred 258 families for direct FN services, primarily for support in accessing services and following through with referrals and recommendations. While nearly a quarter of families needed only one-time support or information, the majority (76%) needed ongoing support and care coordination. An examination of the characteristics of families referred for FN services indicates that most (83%) needed help with accessing or connecting with services for their child. The majority (72%) were enrolled in Medicaid, which is a publicly funded insurance option for eligible individuals with low income, and many (21%) were referred because of family needs or stressors that required specific and immediate support. This suggests that FN may be an especially important service for families with limited resources, who may be facing even greater barriers to accessing healthcare services.

### Limitations and Future Directions

This study describes the results of an initial pilot of a newly developed program, with a focus on evaluation of feasibility, acceptability, and preliminary effects. As such, there are several limitations that should be noted. First, the sample size was relatively small and did not include a control or comparison group. Future studies should employ more rigorous methods, including randomized controlled trial designs, to assess efficacy and effectiveness of the program. In addition, comparative effectiveness studies would be useful to examine how this program compares to more traditional CME approaches (e.g., face-to-face training, workshops, webinars), asynchronous online learning modules, or other models for capacity building.

While the current results are promising in terms of feasibility, acceptability, and provider-level outcomes, additional measures of participant learning and engagement would also be useful for fully examining program effectiveness. Another limitation is that the study did not include direct measures of child or family outcomes. Future research to evaluate this model should include comprehensive assessment of key outcomes, including (but not limited to) age at diagnosis, age at intervention, time between referral and access, development (e.g., communication, social-emotional, adaptive, cognitive functioning), challenging behavior, family satisfaction, family coping, and family self-efficacy. Overall, a multi-method assessment strategy would also provide a more holistic understanding of program effectiveness. As such, future research would benefit from a combination of self-report data, direct observation, and direct assessments of clinician skill and child and family outcomes.

Future research is also needed to examine the cost-effectiveness of the ECHO Autism LINKS model. The ECHO model was designed to “democratize knowledge” by creating a low-cost, accessible, and efficient framework for sharing knowledge with providers in rural and remote areas (Arora, 2019). Infrastructure costs and technology requirements are minimal compared to face-to-face training, allowing participants and hub team members to join from anywhere, saving time and costs associated with travel, and removing geographic barriers. In fact, there are state and federal efforts underway across the country to facilitate sustainability of ECHO and similar models (Expanding Capacity for Health Outcomes Act of 2019; Howe et al., 2017). However, the inclusion of direct FN services into the ECHO model introduces additional costs for implementation. Future research is needed to explore practical barriers and facilitators to implementation and sustainability of this integrated model. This knowledge will be important for informing dissemination and uptake in other contexts.

## Conclusion

Overall, the current results offer preliminary support for the newly developed ECHO Autism LINKS program. Given that children with autism face significant unmet needs for timely, family-centered, coordinated, and comprehensive care (Karpur et al., 2019; Vohra et al., 2014), innovative solutions are needed to address complex healthcare barriers. By combining PCP-focused tele-mentoring with direct FN services, this new model has the potential to provide both PCPs and families with the knowledge and support they need to increase access to care for children with autism. The model also represents a low-cost and highly accessible approach, using a widely available videoconferencing platform to create a virtual learning network, and building the non-specialist autism workforce to improve local access to care. Future large-scale research is needed to evaluate the impact and cost-effectiveness of this program more fully.
